# TOPOIIα and HER-2/neu overexpression/amplification in Barrett’s oesophagus, dysplasia and adenocarcinoma

**DOI:** 10.1111/j.1365-2559.2010.03580.x

**Published:** 2010-07

**Authors:** Elisa Rossi, Vincenzo Villanacci, Gabrio Bassotti, Francesco Donato, Andrea Festa, Gianpaolo Cengia, Salvatore Grisanti, Renzo Cestari

**Affiliations:** 2nd Department of Pathology, University of Brescia – Spedali CiviliBrescia; 1 Gastroenterology & Hepatology Section, Department of Clinical & Experimental Medicine, University of PerugiaPerugia; 2 Institute of Hygiene, Epidemiology and Public Health, Brescia UniversityBrescia, Italy; 3 Digestive Endoscopy, Department of General Surgery, University of Brescia – Spedali CiviliBrescia, Italy; 4 Department of Medical Oncology, Spedali CiviliBrescia, Italy

**Keywords:** adenocarcinoma, Barrett’s oesophagus, Her-2, TopoIIα

## Abstract

Rossi E, Villanacci V, Bassotti G, Donato F, Festa A, Cengia G, Grisanti S & Cestari R (2010) *Histopathology***57,** 81–89 **TOPOIIα and HER-2/neu overexpression/amplification in Barrett’s oesophagus, dysplasia and adenocarcinoma**

**Aims:**

Topoisomerase IIα (*TOPOIIα*) and *HER-2/neu* are chromosome 17q genes coamplified in various cancers; no data exist for Barrett’s oesophagus (BO) and BO adenocarcinoma (ADC). The aim was to investigate gene amplification and protein overexpression of TopoIIα and Her-2/neu in non-dysplastic BO, dysplastic BO, Barrett ADC, and chromosome 17 aneusomy.

**Methods and results:**

Forty-four patients [18 BO, 13 BO with dysplasia (five low-grade dysplasia, eight high-grade dysplasia) and 13 ADC in BO] were evaluated by immunohistochemistry and fluorescence *in situ* hybridization (FISH). Genes (*HER-2/neu* and *TOPOIIα*) and chromosome 17 were evaluated by FISH. Patients with BO, dysplasia and ADC were compared. A significant association was found between TOPOIIα protein overexpression and *TopoIIα* gene amplification, chromosome 17 aneusomy, *HER-2/neu* gene amplification and HER-2 protein overexpression as well as between HER-2 protein and *HER-2/neu* gene, *TopoIIα* gene and aneusomy for chromosome17, and between the genes *TOPOIIα* and *HER-2/neu*. Gene amplification (*HER-2/neu*, *TOPOIIα*), protein overexpression (HER-2/TOPOIIα), and chromosome 17 aneusomy were associated with dysplasia or ADC. Most BO patients showed no amplification/overexpression/aneusomy for the above genes, proteins and chromosome, with no differences between dysplasia and ADC.

**Conclusions:**

HER-2/neu and TOPOIIα amplification/overexpression might discriminate between BO and dysplasia/ADC. Chromosome 17 aneusomy is associated with dysplasia or ADC in BO.

## Introduction

*HER-2/neu* and topoisomerase IIα (*TOPOIIα*) genes are both located on chromosome 17q, and they can be co-amplified in cancer.^1^ Amplification of both genes has been reported in breast, prostatic, gastric, colorectal and pancreatic carcinomas.^1^–^5^*HER-2/neu* oncogene encodes for the human epidermal growth factor receptor 2 and it is a well-known marker of poor prognosis in a variety of tumours.^6^,^7^

There are two isoforms of mammalian topoisomerase II, α and β. DNA topoisomerase II catalyses a transient double-strand DNA break, which allows the passage of another DNA duplex through the break before the strands are resealed. TOPOIIα represents the target enzyme for specific anticancer drugs, such as anthracyclines, commonly used for a variety of both haematological and solid neoplasms, including leukaemias, lymphomas and breast cancer. *In vitro* studies have shown a correlation between the expression level of TOPOIIα in cancer cells and the sensitivity of those cells to topoisomerase inhibitors.^8^,^9^

Some authors have suggested a concordance of *HER-2* and *TOPOIIα* gene amplification in breast cancer,^3^ while others have demonstrated that *TOPOIIα* amplification, identified by fluorescence *in situ* hybridization (FISH), may occur with or without *HER-2* duplication and is often associated with TOPOIIα expression evaluated by immunohistochemistry.^1^

In addition to the fact that amplification of *HER-2* has become a valid biomarker to identify patients with breast cancer who respond to HER-2 protein targeting therapy, several recent clinical trials have found that HER-2-overexpressing breast cancers,^10^ with or without *HER-2* amplification,^11^ are often responsive to anthracycline-based therapies. In fact, it has been proposed that HER-2 amplification in these tumours may be a marker of TOPOIIα amplification.^12^

Recent studies have confirmed that patients with breast cancer with *TOPOIIα* gene amplification are more sensitive to TOPOIIα-based therapy.^13^ How ever, it remains controversial whether gene amplification results in overexpression of the TOPOIIα protein.^9^,^14^,^15^

Adenocarcinoma (ADC) of the oesophagus is currently the cancer with the fastest increasing incidence in the USA, and has replaced squamous cell carcinoma as the most common oesophageal malignancy.^16^,^17^ In fact, an increase in relative and absolute numbers of ADCs of the lower third of the oesophagus has been observed in many Western countries. The most likely explanation for this finding seems to be the increasing prevalence of Barrett’s oesophagus (BO) as a consequence of gastro-oesophageal reflux, which is becoming more common with increasing levels of obesity.

The present study was undertaken to investigate: (i) the role of amplification/overexpression of *TOPOIIα* and *HER-2/neu* genes and proteins, (ii) the association between TOPOIIα amplification/overexpression, HER-2/neu amplification/overexpression and chromosome 17 aneusomy, and (iii) the association between TOPOIIα and HER-2/neu amplification/overexpression and chromosome 17 aneusomy and the presence of BO, low-grade (LGD) or high-grade dysplasia (HGD) and ADC.

## Patients and methods

### Patient selection, clinical and endoscopic evaluation

The clinical records and histological specimens of 44 patients (six women and 38 men, age range 39–89 years) with a confirmed diagnosis of BO were analysed retrospectively. All patients underwent surveillance endoscopy at regular intervals or when clinically indicated at the Digestive Endoscopy Unit of the University of Brescia. Inclusion criteria were: a confirmed histological diagnosis of BO, oesophageal dysplasia (LGD and HGD) and ADC. Overall, specimens were obtained in 32 patients from biopsies and in 12 patients from mucosectomies.

### Pathological evaluation

Immediately after sampling, the specimens were fixed in 10% neutral-buffered formalin for 24 h, routinely processed in paraffin and stained with haematoxylin and eosin (H&E) and Alcian-periodic acid–Schiff for routine histological examination. H&E-stained slides from the resection specimens were evaluated for identification of the steps in cancer progression. ADC and precursor lesions were diagnosed according to the World Health Organization classification,^18^ as previously reported.^19^,^20^ We selected those slides with obvious areas showing BO (100% showed areas with BO not associated with dysplasia), LGD (in >90% of the areas), HGD (in >90%) and ADC (in >90%). The cases of dysplasia were not associated with an invasive carcinoma.

Serial 3-μm sections were cut for FISH and immunohistochemistry, and the first and last sections of each series were stained with H&E. Corresponding areas on sequential sections were thus investigated by the two methods and for both Topo IIα and Her-2/neu.

HER-2 and TOPOIIα status was studied by immunohistochemistry and FISH on paraffin-embedded tissue. Numerical alterations of chromosome 17 [chromosome enumeration probe 17 (CEP17)] were also evaluated by FISH.

#### Immunohistochemistry

HER-2 receptor status was studied using the HercepTest kit (DAKOCytomation, Carpinteria, CA, USA). According to the recommendations of the manufacturer, tissue sections mounted on slides and stored at room temperature (25°C) were stained within 4–6 weeks from sectioning, in order to preserve the antigenicity, then the samples were counterstained with Mayer’s haematoxylin. *HER-2* oncoprotein expression was assessed by two investigators (E.R., V.V.), following the scoring system recommended by the manufacturer’s instructions and the Food and Drug Administration (FDA) guidelines, according to the Hercep Test® criteria.^21^,^22^ Immunoreactivity was scored as follows: 3+, complete and intense membranous reactivity of >10% of tumour cells; 2+, complete but moderate reactivity of >10% of cells; 1+, weak and incomplete reactivity in >10% of cells; and 0, no membranous reactivity, or reactivity in <10% of cells.

To evaluate TOPOIIα protein expression, formalin-fixed, 3 μm thick paraffin-embedded tissues were cut, mounted on charged slides, and dried. For immunohistochemistry, slides were deparaffinized and rehydrated in graded solutions of ethanol and distilled water. Endogenous peroxidase was blocked by incubation with Peroxidase Block and Protein Block (NovoLink Polymer Detection System; Novocastra Laboratories, Newcastle, UK) at room temperature, both for 5 min. The immunohistochemical method involved sequential application of primary antibody to TOPOIIα (H-231, a rabbit polyclonal antibody raised against amino acids 1301–1531 of TOPOIIα of human origin; Santa Cruz Biotechnology, Inc., Santa Cruz, CA, USA) diluted 1:50 for 45 min, then post primary block (NovoLink Polymer Detection System; Novocastra Laboratories) was applied at room temperature for 15 min and a NovoLink Polymer (NovoLink Polymer Detection System; Novocastra Laboratories) for another 15 min. The immunoprecipitate was visualized by treatment with 3′3-diaminobenzidine chromogen (NovoLink Polymer Detection System; Novocastra Laboratories) for 5 min and counterstained by haematoxylin (Dako). Immunoreactivity was considered positive for TOPOIIα when at least 10% of dysplastic/neoplastic cells were stained. We applied the same criteria proposed for the quantification of p53 in endometrial and breast carcinomas.^23^,^24^ All samples were scored quantitatively and qualitatively at ×40 high-power fields (HPF) in every section (Nikon Eclipse E400, Tokyo, Japan) (Area of high-power field = 0.146 mm^2^). TOPOIIα was considered positive when it could be recognized as a nuclear brown stain by immunohistochemistry.

#### FISH

A FDA approved kit for HER-2 evaluation (PathVysion HER-2 DNA Probe Kit; Vysis Inc., Downers Grove, IL, USA) was used, according to the manufacturer’s recommendations. The kit consists of directly labelled fluorescent DNA probes specific for the *Her-2/neu* gene locus (17q11.2-q12), labelled by Spectrum Orange (SO) and a DNA probe specific for the α satellite DNA sequence at the centromeric region of chromosome 17 (17p11.1-q11.1), labelled by Spectrum Green (SG).

*TOPOIIα* gene was labelled by a locus-specific identifier probe for D17Z1 mixed with a probe specific for the centromeric region of chromosome 17 (17p11.1-q11.1) (LSI TOPO2A SO/CEP17 SG; Vysis Inc.). We applied the same protocol and scoring for both HER-2 and TOPOIIα.

Counterstaining of nuclei was performed using 4,6-diamidino-2-phenylindole. A special amplification pattern as *Her-2/neu*‘signal clusters’, usually with >10 confluent signals, was observed, as previously described.^25^,^26^ Although gene amplification as ‘homogeneously stained regions’ was clearly evident, this pattern did not allow precise signal enumeration. Thus, the whole area of each neoplastic lesion present in the tissue section was independently evaluated by two investigators (E.R., V.V.) with fluorescence microscopy (Nikon Optiphot-2, Florence, Italy) equipped with selective filters for the fluorochromes used, in HPF (magnification 600×). FISH images were captured and elaborated using Genikon software (Nikon Instruments S.p.A, Florence, Italy). The *Her-2/neu* gene locus was classified as amplified if there were more than twice the number of red (SO labelling) Her-2/neu signals than green (SG labelling) centromere 17 signals (ratio >2:1) per cell nucleus. The presence of more than two nuclear red signals accompanied by the same number of nuclear green signals was considered to be indicative of aneusomy (in this case polysomy) of chromosome 17 (ratio 1:1). Following these criteria^23^,^27^ the cell population of each HPF was classified as displaying disomy, polysomy or an amplification of the *Her-2/neu* gene.

For *TOPOIIα* and *HER-2* genes, control slides for FISH were bought from the same probe manufacturers (Vysis, FDA approved), while for immunohistochemical analysis for HER-2 protein control slides were provided by the kit used (Dako, FDA approved). For TOPOIIα immunohistochemical analysis (which is the only method not FDA approved) we used as control a slide of breast carcinoma previously shown to be positive with immunohistochemistry and also confirmed positive by FISH.

### Statistical analysis

The associations between *TOPOIIα* gene amplification/TOPOIIα protein overexpression and *HER-2/neu* gene amplification/HER-2 protein overexpression, chromosome 17 aneusomy, and the presence of BO, dysplasia (LGD or HGD) and ADC were evaluated by the usual methods for comparison of proportions. Histology was categorized at three levels: BO, oesophageal dysplasia (which includes LGD and HGD) and ADC. Immunohistochemistry for TopoIIα was categorized at four levels according to quartiles of the percentage: 0–25.0; 25.1–50.0; 50.1–75.0; and 75.1–100%. *P*-values <0.05 (two-tailed tests) were used to reject the null hypothesis. Immunohistochemistry for HER-2 protein was categorized at four levels based on the FDA’s approved scoring system (0, 1, 2, 3); FISH for both *TOPOIIα* and *HER-2/neu* genes was considered positive when amplified and negative when not amplified, and FISH for chromosome 17 was considerate positive or negative in the presence of chromosome aneusomy or disomy, respectively.

### Ethical considerations

Since this was a retrospective study**,** no individual patient identification was done and no study-driven clinical intervention was performed. Thus, a simplified Institutional Review Board approval for retrospective studies was obtained and no patient consent was necessary.

## Results

The characteristics of the 44 subjects included in the study and the results of the overexpression/amplification of the genes investigated are shown in Table 1. *HER-2/neu* gene copy number was higher because clusters were identified (>10 signals), whereas TOPOIIα copy number was lower because it was often possible to count the single spots. No deletions were seen in any of the 44 patients analysed. Almost perfect agreement (43/44, 98%) was found between FISH *TOPOIIα* and *HER-2/neu* gene amplification, with the single exception of subject 22 (*P* < 0.001).

**Table 1:** Characteristics of the patients and results of the gene marker investigation

Case	Age	Sex	Diagnosis	IHC HER2	IHC TOPOIIα (%)	FISH HER2/neu	FISH TOPOIIα	FISH CEP17
1	47	M	BO	1	5	NA	NA	Disomy
2	64	M	BO	1	3	NA	NA	Disomy
3	39	M	BO	0	1	NA	NA	Disomy
4	78	M	BO	0	6	NA	NA	Disomy
5	56	M	BO	1	12.7	NA	NA	Disomy
6	33	M	BO	0	23	NA	NA	Disomy
7	68	M	BO	1	25.3	NA	NA	Disomy
8	61	F	BO	1	33	NA	NA	Disomy
9	66	M	BO	0	3	NA	NA	Disomy
10	69	F	BO	1	55.5	NA	NA	Disomy
11	84	M	BO	0	33	NA	NA	Disomy
12	74	M	BO	1	26.1	NA	NA	Disomy
13	64	M	BO	1	31.2	NA	NA	Disomy
14	48	M	BO	2	40	NA	NA	Disomy
15	82	M	BO	0	8	NA	NA	Disomy
16	67	M	BO	1	40.8	NA	NA	Disomy
17	52	M	BO	0	14.44	NA	NA	Disomy
18	75	M	BO	0	3.20	NA	NA	Disomy
19	72	F	LGD	0	35	NA	NA	Disomy
20	56	M	LGD	0	13	NA	NA	Disomy
21	74	F	LGD	3	96	A	A	Disomy
22	76	M	LGD	3	37	A	NA	Aneusomy
23	77	M	LGD	2	50.2	NA	NA	Disomy
24	59	M	HGD	1	58	NA	NA	Aneusomy
25	89	M	HGD	0	38	NA	NA	Disomy
26	76	M	HGD	3	60	A	A	Aneusomy
27	48	M	HGD	1	44.5	NA	NA	Disomy
28	85	M	HGD	3	90	A	A	Disomy
29	53	M	HGD	3	87	A	A	Aneusomy
30	82	M	HGD	3	100	A	A	Disomy
31	51	M	HGD	3	37	NA	NA	Disomy
32	69	M	ADC	1	53	NA	NA	Aneusomy
33	75	M	ADC	3	100	A	A	Disomy
34	89	M	ADC	1	42.3	NA	NA	Aneusomy
35	67	F	ADC	1	84	NA	NA	Aneusomy
36	83	M	ADC	2	49	NA	NA	Aneusomy
37	78	M	ADC	2	43	NA	NA	Aneusomy
38	80	M	ADC	1	49	NA	NA	Disomy
39	71	F	ADC	3	80	A	A	Aneusomy
40	58	M	ADC	3	87	A	A	Aneusomy
41	76	M	ADC	1	65	NA	NA	Disomy
42	68	M	ADC	3	65	A	A	Disomy
43	77	M	ADC	2	97	NA	NA	Aneusomy
44	73	M	ADC	3	75	A	A	Disomy

M, male; F, female; BO, Barrett’s oesophagus; LGD, low-grade dysplasia; HGD, high-grade dysplasia; ADC, adenocarcinoma; IHC, immunohistochemistry; FISH, fluorescence *in situ* hybridization; A, amplified; NA, not amplified; CEP, chromosome enumeration probe.

IHC Her2: values from 0 to 3 were attributed according to Food and Drug Administration instructions.

A statistically significant association was found between immunohistochemical TOPOIIα protein overexpression and FISH *TOPOIIα* gene amplification (*P* < 0.001), FISH *HER-2/neu* gene amplification (*P* < 0.001), immunohistochemical HER-2 protein overexpression (*P* < 0.001) and FISH chromosome 17 aneusomy (*P* = 0.03). A strong association was also found between immunohistochemical HER-2 protein overexpression and FISH *HER-2/neu* gene amplification (*P* < 0.001), FISH *TOPOIIα* gene amplification (*P* < 0.001) and FISH aneusomy for chromosome17 (*P* = 0.02). No association was found between FISH chromosome 17 aneusomy and either FISH *HER-2/neu* or *TOPOIIα* gene amplification (*P* > 0.1 for both).

**Figure 1:**
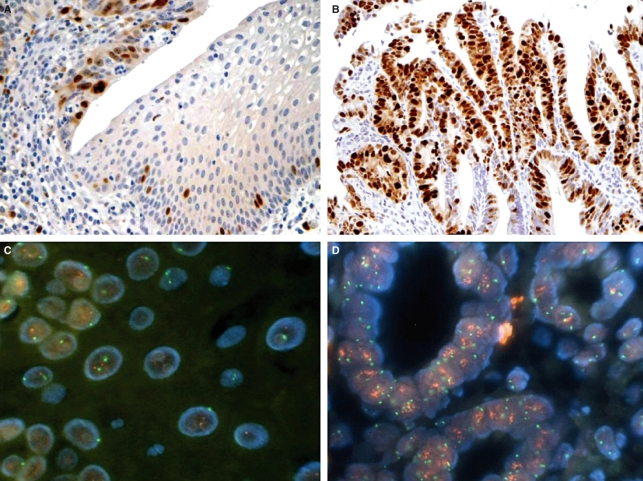
**A,B,** Immunohistochemistry for TOPOIIα. **A,** right part: normal oesophagus largely negative for TOPOIIα; only in the basal layer is it possible to recognize some positive cells. On the left is an area of dysplasia where the positivity increases (case 22, Table 1). **B,** Area of high-grade dysplasia, where the cells are positive for TOPOIIα (case 30, Table 1). **C,D,** Fluorescence *in situ* hybridization for TOPOIIα. **C,** A normal oesophagus displays two signals for TOPOIIα (red spots) and for chromosome17 (green spots). **D,** Low-grade dysplasia with gene amplification (patient 22, Table 1).

**Figure 2:**
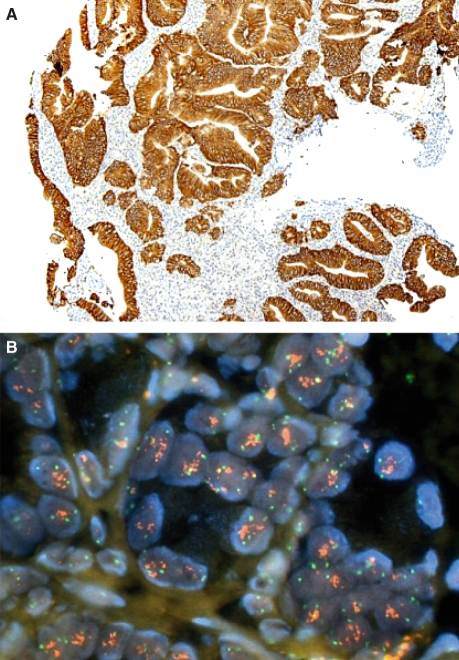
**A,B,** Case 29. HER-2 analysed respectively by immunohistochemistry and fluorescence *in situ* hybridization. **A,** All the areas of dysplasia are positive for the membranous stain which identifies HER-2 receptor. **B,** The same area of dysplasia. *HER-2* gene amplification (red spots) is strong and shows typical clusters; chromosome17 aneusomy is present in all the nuclei with more than two green signals.

Table 2 shows the distribution of the three groups of subjects according to each gene/protein assessment and chromosome aneusomy/disomy. Gene amplification (FISH *HER-2/neu* and *TOPOIIα*), protein overexpression (HER-2, TOPOIIα) and chromosome 17 aneusomy were all associated with the presence of dysplasia or ADC with respect to BO (*P* < 0.05 for each comparison). Among subjects with BO, none showed HER-2/neu or TOPOIIα amplification or chromosome 17 aneusomy, and almost none had moderate to complete membranous immunoreactivity for HER-2 protein expression (immunohistochemistry) (score 2 or 3) or >50% TOPOIIα protein expression (immunohistochemistry) (quartiles 3–4). On the other hand, 30–61% of subjects with dysplasia or ADC showed gene amplification and protein overexpression for both HER-2/neu or TOPOIIα and chromosome 17 aneusomy. No significant differences in positivity were seen between dysplasia and ADC for any the markers analysed. Representative images are shown in Figures 1 and 2.

**Table 2:** Distribution of subjects according to gene amplification, protein expression and histology

	Histology				
	BO	Dysplasia (LGD-HGD)	ADC	All pathologies	
Chromosome 17 genes/proteins	No. (%)	No. (%)	No. (%)	No. (%)	*P*-value*
Total subjects	18 (100)	13 (100)	13 (100)	44 (100)	
HER-2 protein (IHC)					
0	8 (44.4)	3 (23.1)	0 (–)	11 (25.0)	0.001
1	9 (50.0)	3 (23.1)	5 (38.5)	17 (38.6)	
2	1 (5.6)	1 (7.7)	3 (23.1)	5 (11.4)	
3	0 (–)	6 (46.2)	5 (38.5)	11 (25)	
*HER-2/neu* gene (FISH)					
0 (NA)	18 (100)	7 (53,8)	8 (61.5)	33 (75)	0.002
1 (A)	0 (–)	6 (46)	5 (38.4)	11 (25)	
Chromosome 17 (FISH)					
0 (Disomy)	18 (100)	9 (69.2)	5 (38.5)	32 (72.7)	0.004
1 (Aneusomy)	0 (–)	4 (30.8)	8 (61.5)	12 (27.3)	
TopoIIα protein (IHC)					
1 (1.0–25.0%)	10 (55.5)	1 (7.7)	0 (–)	11 (25)	0.001
2 (25.1–50%)	7 (38.9)	5 (38.44)	4 (30.8)	16 (36.3)	
3 (50.1–75%)	1 (5.55)	3 (23)	4 (30.8)	8 (18.1)	
4 (75.1–100%)	0 (–)	4 (30.8)	5 (38.44)	9 (20.4)	
*TopoIIα* gene (FISH)					
0 (NA)	18 (100)	8 (61.5)	8 (61.55)	34 (77.3)	0.004
1 (A)	0 (–)	5 (38.4)	5 (38.44)	10 (22.7)	

BO, Barrett’s oesophagus; LGD, low-grade dysplasia; HGD, high-grade dysplasia; ADC, adenocarcinoma; IHC, immunohistochemistry; FISH, fluorescence *in situ* hybridization; A, amplified; NA, not amplified.

* Exact tests for the comparison among proportions of patients with BO, dysplasia and ADC for each gene overexpression/amplification.

With the single exception of case 22, all the cases of dysplasia and ADC showed co-amplification of *TOPOIIα* and *HER-2/neu* genes (>50%). *HER-2/neu* gene amplification was higher then *TOPOIIα* (Figure 1D) because of the clustering (>10 signals) (Figure 2B), as also reported in previously.^3^,^4^,^28^ No deletions were seen in any of the 44 patients analysed. There was no significant association between protein/gene overexpression/amplification and age or gender (data not shown).

## Discussion

Topoisomerases are nuclear enzymes that regulate cellular processes such as replication and transcription; in addition, these enzymes represent a major molecular target for drugs of the anthracycline class or chemotherapeutic agents.^9^ Clinical and *in vitro* evidence supports the concept that in breast cancer the association between HER-2 amplification and response to anthracycline-based chemotherapy is not a direct effect of HER-2 overexpression but the result of co-amplification of the *TOPOIIα* gene.^29^ Some studies have shown that increased expression of TOPOIIα in BO is associated with HGD,^30^ as well as the fact that HER-2 is overexpressed in oesophageal ADC^31^ and this overexpression may predict early transition from dysplasia to ADC in BO.^32^ However, to the best of our knowledge the expression of TOPOIIα and HER-2 has not been previously investigated in BO patients.

In the present study we examined HER-2/neu and TOPOIIα gene/protein by FISH and immunohistochemistry, respectively, and we also investigated chromosome 17 status and histological features (BO, LGD, HGD and ADC).

In agreement with previous studies on breast carcinomas,^3^ we confirmed that TOPOIIα is rarely amplified in the absence of HER-2 amplification, that it is co-amplified with HER-2 in dysplasia (LGD and HGD) and ADC, and that the *HER-2* gene copy number, because of clustering, was higher than the *TOPOIIα* copy number. Moreover, statistically significant associations were found between gene amplification and protein expression for both HER-2 and TOPOIIα, between *HER-2* and *TOPOIIα* gene amplification and between HER-2 and TOPOIIα protein expression, confirming the results of previous studies regarding pancreatic and gastric cancers.^2^,^4^ Chromosome 17 aneusomy was found to be associated with TOPOIIα protein overexpression but not with each gene amplification. No deletions for TOPOIIα and/or HER-2/neu were seen in any of our patients, whereas monoallelic deletions were found in other pathological conditions, i.e. breast cancer.^3^

We investigated the associations between the above-mentioned markers and pathological findings. We found HER-2/TOPOIIα gene amplification/protein overexpression and chromosome 17 aneusomy (polysomy) in a high proportion of patients with dysplasia (LGD and HGD) or ADC, but in almost none of the patients with BO, suggesting involvement of these factors in cancer development. It is well known that BO is associated with gastrointestinal acid reflux, and it has been suggested that acid reflux could introduce mutations in oesophageal cells due to acid pH-induced DNA damage.^33^ However, acid reflux also causes inflammatory responses known to contribute to carcinogenesis.^34^

Recent clinical studies have shown that *TOPOIIα* gene amplification is a more specific predictor than TOPOIIα expression assessed by immunohistochemistry and *HER-2* gene amplification for clinical response to TOPOIIα inhibitors in breast cancer.^35^ For this reason, FISH testing for TOPOIIα status in addition to HER-2 evaluation may be useful in the characterization of Barrett’s pathology and progression to dysplasia. Furthermore, ascertaining TOPOIIα status might be useful to select patients for combination therapy: a trial could be designed to investigate whether patients with TOPOIIα amplification may be treated with HER-2 targeting drug together with a cytotoxic drug, such as TOPOIIα-inhibitor.

In conclusion, these findings suggest that the investigation of these markers might be useful in characterizing the evolution from BO to dysplasia and ADC. These potential markers might also contribute to deciding alternative therapeutic approaches, as suggested by some preliminary data.^36^

## Abbreviations:

ADC: adenocarcinoma
BO: Barrett’s oesophagus
CEP17: chromosome enumeration probe 17
FDA: Food and Drug Administration
FISH: fluorescence *in situ* hybridization
H&E: haematoxylin and eosin
HGD: high-grade dysplasia
HPF: high-power field
LGD: low-grade dysplasia
SG: Spectrum Green
SO: Spectrum Orange
TOPOIIα: topoisomerase IIα
